# One- to 10-year Status Epilepticus Mortality (SEM) score after 30 days of hospital discharge: development and validation using competing risks analysis

**DOI:** 10.1186/s12883-019-1540-y

**Published:** 2019-12-01

**Authors:** Prapassara Sirikarn, Porjai Pattanittum, Somsak Tiamkao

**Affiliations:** 1 Department of Epidemiology and Biostatistics, Faculty of Public Health, Khon Kaen University, Khon Kaen, Thailand; 2 Integrated Epilepsy Research Group, Khon Kaen University, Khon Kaen, Thailand; 3 Division of Neurology, Department of Medicine, Faculty of Medicine, Khon Kaen University, Khon Kaen, Thailand

**Keywords:** Status epilepticus, Score, Predictive model, Long-term, Mortality

## Abstract

**Background:**

Status epilepticus (SE) is an emergency neurological disorder that affects quality of life and is associated with high mortality risk**.** Three scores have been developed to predict the risk of in-hospital death, but these scores are poor discrimination of mortality after discharge**.** This study aimed to develop and validate a simple risk score for long-term mortality in SE patients.

**Methods:**

This retrospective cohort study was conducted using SE patient data collected from Thailand’s Universal Coverage Scheme database between the fiscal years of 2005 and 2015 and followed-up to 2016. Patients who died in hospital or within 30 days after discharge were excluded. Data were divided at random into either a derivation or validation set. A proportional hazards model for the sub-distribution of competing risks was fitted with backward stepwise method. The coefficients from the model were used to develop a point-based scoring system. The discrimination ability of the model was evaluated using a time-dependent receiver operating characteristic (ROC) curve.

**Results:**

A total of 20,792 SE patients (with ages ranging from the first day of life to 99 years at first admission) were randomly separated into two groups: 13,910 in the development group and 6882 in the validation group. A sub-distribution hazard model was used to determine nine predictors to be included in the final model, which was, in turn, used to develop the scoring system: age (0–19 points), male (two points), brain tumor (12 points), stroke (three points), cancer (11 points), diabetes (three points), chronic kidney disease (five points), pneumonia (five points), and urinary tract infection (four points). The possible total score ranged from zero to 64 and the cumulative incidence function was used to determine the probability of mortality associated with each total score within the first 10 years after the first admission. The area under the ROC curve (AUC) of the first to last time point ranged from 0.760 to 0.738.

**Conclusion:**

A nine-factor risk score for predicting 10-year mortality in SE patients was developed. Further studies should focus on external validity and including a range seizure types and duration of seizure as the predictors.

## Background

Status epilepticus (SE) is an emergency neurological disorder that affects patients’ quality of life and is associated with high mortality risk. A recent meta-analysis found that the pooled crude annual incidence rate of SE was 12.6 per 100,000 person-years (95% confidence interval [CI]: 10.0 to 15.3) and the case fatality rate was 14.9% (95% CI: 11.7 to 18.7%) [1].

There are several factors that contribute to the risk of death in SE patients, but the key factors are age, duration of SE, and etiology [2, 3]. Moreover, previous studies found that comorbidities and complications were also major factors associated with mortality, for example, brain tumors, central nervous system (CNS) infection, septicemia, pneumonia, and shock [4–7]. Because death is a crucial outcome in SE, identifying the risk of death in patients with this condition can allow physicians to optimally manage their patients’ care on an individual basis. Risk scores have been developed to assess the risk of death in SE based on several important factors in order to assist health care professionals in the therapeutic decision-making process.

Currently, there are three available scores to predict a patient’s risk of in-hospital death. The most commonly used score is the Status Epilepticus Severity Score (STESS). This score was developed based on four predictors: consciousness, seizure type, age, and history of previous seizure [8]. Another is the Epidemiology-Based Mortality Score in Status Epilepticus (EMSE), which takes epidemiological data, including etiology, age, electroencephalogram (EEG), and comorbidity, into account [9]. Finally, the modified Status Epilepticus Severity Score (mSTESS) was developed from the STESS to more accurately predict mortality at discharge by including the patient’s Modified Rankin Scale (mRS) in with the other predictors used in the STESS [10].

The STESS has also been applied to predict long-term mortality in SE patients, but was found to be a poor discrimination of mortality after discharge (the area under the receiver operating characteristic curve [AUC] was 0.676 (95%CI: 0.516 to 0.833)) [11].

Most of the published studies mentioned above only followed SE patients through their hospital stay and lacked data predicting long-term mortality. The outcome of interest in this study is time-to-death after 30 days of hospital discharge. It was defined as time from the patient’s first admission with a primary diagnosis of SE to death which including both direct (e.g. seizure, epilepsy, SE) and indirect (e.g. SE complications, suicide, accidents, or underlying diseases [12]) causes of deaths by taking into account for the competing risks (e.g. senility, cardiovascular collapse). Thus, this study aimed to develop and validate a score to predict long-term mortality in SE patients that accounts for the presence of competing risks in order to avoid overestimating the of probability of death in SE, which tends to be a flaw in the conventional statistical methods that are used for this purpose [13, 14].

## Methods

### Design and setting

This was a retrospective cohort study. Data for this study was retrieved from the Thai Universal Coverage Scheme electronic database that recorded the information of over 75% of Thai citizens who admitted to hospitals within Universal Coverage Scheme. Data between the fiscal years of 2005 and 2015 (October 1, 2004 to September 30, 2015) were used and followed up for 1 year (until September 30, 2016).

### Patients

Patients were eligible for inclusion in this study if their data were in the Thai Universal Coverage Scheme database and they were admitted to hospitals with a primarily diagnosis of SE based on the guidelines described in the International Statistical Classification of Diseases and Related Health Problems – 10th Revision (ICD-10) code G41 (Status epilepticus). The SE diagnosis was followed by the guideline of the International League Against Epilepsy in each version over past 10 years. There were no restrictions based on age, sex, or SE type. Eligibility was limited to those patients who had not died in hospital (discharge status of death) or within 30 days after discharge. Patients with incomplete data regarding their date of birth, date of admission, date of death, or cause of death were also excluded. All patient’ data were anonymized and de-identified prior to extraction and analysis.

### Study variables

The outcome in this study was time from the patient’s first admission with a primary diagnosis of SE to death (including both direct and indirect causes). Deaths from the following causes according to the patients’ death certificates were considered to be event of interest: (a) seizure, epilepsy, SE, (b) accident, suicide, (c) SE complications (e.g. pneumonia, septicemia), and (d) comorbidities (e.g. cancer, diabetes mellitus; Additional file 1: Figure S1). Competing risks events were senility, cardiovascular collapse, etc. (Additional file 1: Table S1 provides the details regarding event of interest and competing events in this study). Patients who were still alive at the end of the study (September 30, 2016) were censored data.

Predictors in this study included baseline demographic characteristics (sex and age at first admission), comorbidities, and complications (occurred during the follow-up period). ICD-10 codes were utilized to identify comorbidities and complications (Additional file 1: Table S2).

### Statistics

The eligible study subjects were randomly split into a derivation set and a validation set at a 2:1 ratio. Characteristics of patients in both sets were reported as frequency and percentage for categorical data and median with minimum and maximum value for continuous data. A sub-distribution hazard function was used to develop a predictive model with competing risks [15, 16]. Sensitivity analysis was performed based on selection algorithms, and the modified Bayesian information criterion for competing risks was used to select predictors for the final model [17]. The coefficients in the final sub-distribution hazard model were used as a scoring system that followed by the method of Austin [14]. Although the concordance index (c-index) has been widely used to evaluate the performance of predictive models with time-to-event outcomes, the c-index tends to be misleadingly high when used to predict t-year risk. Thus, we deemed the time-dependent receiver operating characteristic (ROC) curve to be more appropriate to assess differences at each year point [18, 19]. Statistical analyses were performed using the R statistical programming language [20] with the “crrstep” package [21] to select predictors, the “cmprsk” package [22] for fitting the sub-distribution hazard model, and “riskRegression” package [23] for assessing the time-dependent ROC.

## Results

**Fig. 1:**
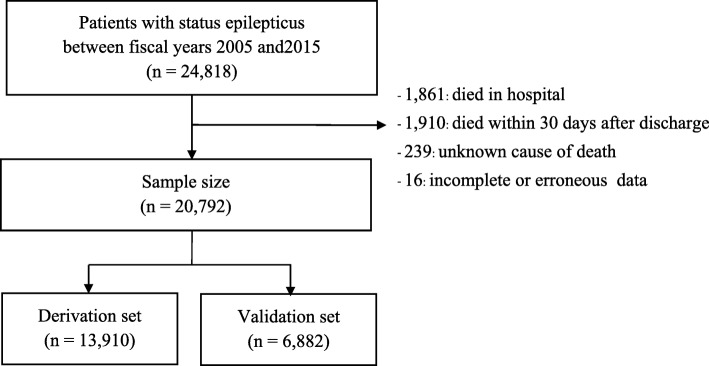
The inclusion flowchart

Among the 24,818 SE patients who were admitted to hospitals included in the Thai Universal Coverage Scheme database between the fiscal years of 2005 and 2015, 4026 cases were excluded because the patient died in hospital, died within 30 days after discharge, had an unknown cause of death, or had missing or erroneous data. Of the remaining 20,792 SE patients, 13,910 were randomly allocated to the derivation set and 6882 to the validation set (Fig. 1). There were 3594 cases of death due to direct and indirect causes (derivation = 2446, validation = 1148), and 1073 died due to competing risks events (derivation = 701, validation = 372). The median age of all participants was 31 years (range: 0–99), and 64% were male. Characteristics of patients in the derivation set and those in the validation set were comparable (Table 1).

**Table 1:** Characteristics of admitted SE patients on the national database during fiscal year 2005–2015

Characteristics	Derivation set		Validation set	
	Number	(%)	Number	(%)
Male*	8836	(63.5%)	4437	(64.5%)
Age at first admission (years)*^,^**	31 (0: 99)		32 (0: 94)	
Status epilepticus (ICD-10 code)				
Convulsive SE (G41.0, G41.9)	13,417	(96.5%)	6658	(96.7%)
Non-convulsive SE (G41.1, G41.2)	266	(1.9%)	121	(1.8%)
Other SE (G41.8)	227	(1.6%)	103	(1.5%)
Brain tumor*	93	(0.7%)	44	(0.6%)
Stroke*	1682	(12.1%)	826	(12.0%)
Epilepsy	1290	(9.3%)	632	(9.2%)
CNS infection	256	(1.8%)	132	(1.9%)
Cancer*	179	(1.3%)	68	(1.0%)
Diabetes*	794	(5.7%)	388	(5.6%)
Hypertension*	1381	(9.9%)	675	(9.8%)
Chronic kidney disease*	250	(1.8%)	125	(1.8%)
Heart diseases*	412	(3.0%)	199	(2.9%)
Ischemic heart disease*	117	(0.8%)	73	(1.1%)
Shock*	122	(0.9%)	65	(0.9%)
Septicemia*	434	(3.1%)	221	(3.2%)
Hypoglycemia*	247	(1.8%)	124	(1.8%)
Pneumonia*	1672	(12.0%)	827	(12.0%)
Respiratory failure*	2293	(16.5%)	1157	(16.8%)
Acute renal failure*	251	(1.8%)	107	(1.6%)
Urinary tract infection*	597	(4.3%)	267	(3.9%)

*candidate predictors in the initial model (with *P* value < 0.20 in the univariate model)

**Median (Min:Max)

### Developing a predictive model from the derivation set

There were 17 candidate predictors that were included in the initial crude analysis with sub-distribution modelling (Table 1; Items marked with an asterisk [*]). After application of the forward or backward stepwise procedure, nine predictors remained relevant in the final model (Table 2).

**Table 2:** Predictors associated with long-term mortality in SE^a^ according to the sub-distribution hazard model

Predictors	Unadjusted				Adjusted			
	β	SHR	95%CI	*p*-value	β	SHR	95%CI	*p*-value
Age at first admission (years)	0.0256	1.03	1.02–1.03	< 0.001	0.0211	1.02	1.02–1.02	< 0.001
Male	0.2432	1.28	1.17–1.39	< 0.001	0.2458	1.28	1.17–1.40	< 0.001
Brain tumor	1.3641	3.91	2.92–5.24	< 0.001	1.2451	3.47	2.52–4.79	< 0.001
Stroke	1.0103	2.75	2.50–3.02	< 0.001	0.2822	1.33	1.19–1.48	< 0.001
Cancer	1.6646	5.28	4.23–6.60	< 0.001	1.2065	3.34	2.58–4.33	< 0.001
Diabetes	1.0242	2.78	2.45–3.16	< 0.001	0.3515	1.42	1.23–1.64	< 0.001
Chronic kidney disease	1.2753	3.58	2.91–4.40	< 0.001	0.5136	1.67	1.33–2.11	< 0.001
Pneumonia	0.3720	1.45	1.30–1.62	< 0.001	0.4763	1.61	1.43–1.81	< 0.001
Urinary tract infection	0.8843	2.42	2.09–2.80	< 0.001	0.3946	1.48	1.27–1.74	< 0.001

^a^both direct and indirect causes

### Developing a risk score from the predictive model using competing risks analysis

Of the nine predictors included in the final model, age at first admission, a continuous predictor, was classified into age categories at five-year intervals (Table 3). After following the steps required for the point-based scoring system, scores were assigned to all predictors. The possible total score ranged from zero to 64 (Table 4). The total score was used to determine the probability of event occurrence based on the cumulative incidence function for each year point (Table 5).

**Table 3:** Point-based risk scoring system for long-term mortality in SE^a^

Predictors^a^	Categories	Ref. value	β_i_^b^	β_i_ (W_ij_-W_iREF_)	Points^c^
Age at first admission	0–4	2 = W_iREF_	0.0211	0	0
	5–9	7		0.106	1
	10–14	12		0.211	2
	15–19	17		0.317	3
	20–24	22		0.422	4
	25–29	27		0.528	5
	30–34	32		0.633	6
	35–39	37		0.739	7
	40–44	42		0.844	8
	45–49	47		0.950	9
	50–54	52		1.055	10
	55–59	57		1.161	11
	60–64	62		1.266	12
	65–69	67		1.372	13
	70–74	72		1.477	14
	75–79	77		1.583	15
	80–84	82		1.688	16
	85–89	87		1.794	17
	90–94	92		1.899	18
	95–99	97		2.005	19
Sex	Female	0 = W_iREF_	0.2458	0	0
	Male	1		0.246	2
Brain tumor	No	0 = W_iREF_	1.2451	0	0
	Yes	1		1.245	12
Stroke	No	0 = W_iREF_ 1	0.2822	0	0
	Yes			0.282	3
Cancer	No	0 = W_iREF_	1.2065	0	0
	Yes	1		1.207	11
Diabetes	No	0 = W_iREF_	0.3515	0	0
	Yes	1		0.352	3
Chronic kidney disease	No	0 = W_iREF_	0.5136	0	0
	Yes	1		0.514	5
Pneumonia	No	0 = W_iREF_	0.4763	0	0
	Yes	1		0.476	5
Urinary tract infection	No	0 = W_iREF_	0.3946	0	0
	Yes	1		0.395	4

^a^both direct and indirect causes

^b^The coefficients form the final sub-distribution hazard model

^c^points were computed by β_i_ (W_ij_-W_iREF_)/(5β_age_) and rounded into integer value

**Table 4:** Risk score for long-term mortality in SE^a^

Predictors	Scores
Age at first admission (years)	
0–5	0
6–10	1
11–15	2
16–20	3
21–25	4
26–30	5
31–35	6
36–40	7
41–45	8
46–50	9
51–55	10
56–60	11
61–65	12
66–70	13
71–75	14
76–80	15
81–85	16
86–90	17
91–95	18
96–100	19
Male	2
Brain tumor	12
Stroke	3
Cancer	11
Diabetes	3
Chronic kidney disease	5
Pneumonia	5
Urinary tract infection	4

^a^both direct and indirect causes

**Table 5:** Predicted mortality rates in SE^a^ with individual score totals

Score points	Predicted mortality (%)									
	1 yr	2 yrs	3 yrs	4 yrs	5 yrs	6 yrs	7 yrs	8 yrs	9 yrs	10 yrs
0	2.1	3.2	4.2	5.3	6.2	7.0	7.8	8.5	9.2	9.8
1	2.3	3.5	4.7	5.8	6.9	7.8	8.6	9.4	10.2	10.9
2	2.5	3.9	5.2	6.5	7.6	8.6	9.5	10.4	11.2	12.0
3	2.8	4.3	5.8	7.2	8.4	9.5	10.5	11.5	12.4	13.3
4	3.1	4.8	6.4	7.9	9.3	10.5	11.6	12.6	13.7	14.6
5	3.5	5.3	7.1	8.8	10.3	11.6	12.8	13.9	15.1	16.1
6	3.8	5.9	7.8	9.7	11.4	12.8	14.1	15.4	16.6	17.7
7	4.3	6.6	8.7	10.7	12.6	14.2	15.6	16.9	18.3	19.5
8	4.7	7.3	9.6	11.8	13.9	15.6	17.1	18.6	20.1	21.4
9	5.2	8.0	10.6	13.1	15.3	17.2	18.8	20.5	22.1	23.5
10	5.8	8.9	11.7	14.4	16.8	18.9	20.7	22.5	24.2	25.7
11	6.4	9.8	12.9	15.9	18.5	20.8	22.7	24.6	26.5	28.2
12	7.1	10.9	14.2	17.5	20.4	22.8	24.9	27.0	29.0	30.8
13	7.9	12.0	15.7	19.2	22.4	25.0	27.3	29.5	31.6	33.5
14	8.7	13.2	17.3	21.1	24.5	27.3	29.8	32.2	34.5	36.5
15	9.6	14.6	19.0	23.2	26.8	29.9	32.5	35.0	37.5	39.6
16	10.6	16.1	20.9	25.4	29.3	32.6	35.4	38.1	40.7	42.9
17	11.7	17.7	22.9	27.8	32.0	35.5	38.5	41.3	44.0	46.4
18	12.9	19.5	25.1	30.4	34.9	38.5	41.7	44.7	47.5	49.9
19	14.3	21.4	27.5	33.1	37.9	41.8	45.1	48.2	51.2	53.7
20	15.7	23.5	30.0	36.0	41.1	45.2	48.6	51.8	54.9	57.5
21	17.3	25.7	32.7	39.1	44.5	48.7	52.3	55.6	58.7	61.3
22	19.1	28.1	35.7	42.4	48.0	52.4	56.1	59.4	62.6	65.2
23	20.9	30.7	38.7	45.8	51.7	56.2	59.9	63.3	66.5	69.0
24	23.0	33.5	42.0	49.4	55.4	60.0	63.8	67.2	70.3	72.8
25	25.2	36.4	45.4	53.1	59.2	63.9	67.7	71.0	74.1	76.5
26	27.6	39.5	48.9	56.9	63.1	67.8	71.5	74.7	77.7	80.0
27	30.1	42.8	52.6	60.7	67.0	71.6	75.2	78.3	81.1	83.3
28	32.8	46.3	56.4	64.6	70.8	75.3	78.8	81.7	84.3	86.3
29	35.8	49.9	60.3	68.5	74.6	78.9	82.1	84.9	87.2	89.0
30	38.8	53.6	64.1	72.3	78.2	82.2	85.2	87.7	89.8	91.4
31	42.1	57.4	68.0	76.0	81.6	85.3	88.1	90.3	92.1	93.5
32	45.5	61.2	71.8	79.5	84.7	88.1	90.6	92.5	94.1	95.2
33	49.1	65.1	75.5	82.8	87.6	90.6	92.8	94.4	95.7	96.6
34	52.7	69.0	79.1	85.9	90.2	92.8	94.6	95.9	96.9	97.6
35	56.5	72.8	82.4	88.6	92.4	94.6	96.1	97.1	97.9	98.4
36	60.4	76.4	85.5	91.1	94.3	96.1	97.3	98.1	98.7	99.0
37	64.3	79.9	88.3	93.2	95.9	97.3	98.2	98.8	99.2	99.4
38	68.1	83.2	90.8	94.9	97.1	98.2	98.8	99.2	99.5	99.7
39	> 71.9	> 86.2	> 92.9	> 96.4	> 98.0	> 98.8	> 99.3	> 99.6	> 99.7	> 99.8

^a^both direct and indirect causes

### Validating the predictive model from both the derivation and validation sets

The AUCs in the derivation set were 0.760, 0.745, 0.742, 0.734, 0.741, 0.739, 0.741, 0.743, 0.742, and 0.738 at each year from years one to 10, respectively. In the validation set, the AUCs for each year from years one to 10 were 0.761, 0.743, 0.740, 0.734, 0.722, 0.725, 0.733, 0.737, 0.733, and 0.740 (Table 6).

**Table 6:** Discrimination in the derivation and validation sets

Years	Derivation set		Validation set	
	AUC	95%CI	AUC	95%CI
1	0.760	0.743–0.777	0.761	0.736–0.785
2	0.745	0.730–0.760	0.743	0.722–0.764
3	0.742	0.725–0.759	0.740	0.719–0.760
4	0.734	0.716–0.752	0.734	0.714–0.755
5	0.741	0.717–0.765	0.722	0.699–0.746
6	0.739	0.706–0.773	0.725	0.697–0.753
7	0.741	0.697–0.785	0.733	0.699–0.768
8	0.743	0.685–0.801	0.737	0.695–0.780
9	0.742	0.665–0.819	0.733	0.677–0.789
10	0.738	0.639–0.836	0.740	0.668–0.812

## Discussion

This study developed and internally validated a new 10-year prediction score for mortality in SE patients after their first admission using a competing risks approach. This simple prediction risk score relied on demographic data, comorbidities, and complications (age, sex, brain tumor, stroke, cancer, diabetes, chronic kidney disease, pneumonia, and urinary tract infection). These finding should be considered with caution, because this study focused on the long-term mortality (outside hospital), we cannot guarantee that the indirect causes of death (accident, suicide, SE complication, and comorbidity) were due to SE. We found that age was a major predictor of mortality in SE patients, which is in accordance with results found using the STESS and EMSE [8, 9]. Similarly, brain tumor, stroke, diabetes, and chronic kidney disease have been identified as predictors using the EMSE [9]. Because this study focused on long-term mortality in SE patients, gender, cancer, pneumonia, and urinary tract infection scores differed from those found in previous studies. However, prior articles have found clear associations between these predictors and mortality in SE patients [4, 6, 7, 24], except the urinary tract infection which shown as a protective factor in previous study [7]. This is due to the difference group of patients – inpatients and after discharge within 30 days, and the outcome – all-cause mortality in SE.

The discriminatory capability of the proposed prediction model using the AUC at each year from years one to 10 were all over 70% for both the derivation and validation sets, which is slightly lower than that of the STESS (AUC = 0.760; in-hospital death) [8]. However, a recent study examining long-term mortality utilized the STESS and found that it had a poor discriminatory ability (AUC = 0.676) [11]. Another study found that the discrimination capability of the EMSE (AUC = 0.902; in-hospital death) [9] decreased when it was applied to 30-day mortality (AUC = 0.832) [25]. Although the performance of our model was generally consistent, the 95% CI of the AUC was wider in the later periods than earlier on due to the lower death rates in the later years [26].

There were three major strengths of this study as a result of our use of Thailand’s Universal Coverage Scheme dataset to develop the risk score. First, it allowed us to access a large sample size consisting of patient data from multicenter and follow them over 10 years. Second, as the database contains the information of patients in all age ranges, the score we developed can be generalized to SE patients of all ages. Lastly, the Universal Coverage Scheme database is connected to that of the Ministry of the Interior, which allowed us to follow up on all patients. However, the death of patients in this study is out-of-hospital mortality. If the patients died with natural causes, the definitive cause of death would not be identified because they were not performed by autopsy, even so, the sensitivity analysis found that the AUCs of first to last time points ranged from 0.722 to 0.692 that decreased by 0.038 to 0.046 when the patients who died with comorbidities were excluded. Furthermore, most of the healthcare units lacked the capability to perform EEG (difficult to specify the type of seizure). Therefore, most of patients in this study were diagnosed with convulsive SE and our study included all type of SE in order to avoid misclassification of SE types which be lead to underestimation or overestimation. Moreover, the database did not contain information about duration of seizure and etiology, which are the important predictors associated with death in SE patients. Thus, further studies should include seizure types, duration of seizure, and etiology as predictors. In addition, we lacked of external validation due to this database containing insufficient longitudinal data. Therefore, external validation should be performed in order to evaluate the generalizability of this study.

## Conclusions

In this study, we developed the first simple clinical scoring system that considers competing risks to predict long-term mortality after first admission in SE patients. The score is based on demographic data, comorbidities, and complications (age, sex, brain tumor, stroke, cancer, diabetes, chronic kidney disease, pneumonia, and urinary tract infection). The AUCs of the first to last time point ranged from 0.760 to 0.738. This user-friendly score requires only simple information of patient that contained in the medical records; where clinical or laboratory data was not available. In addition, the risk score can assist the patients to realize their own risk and helps clinicians in their decision-making process about long-term plan for treatment or reduce risk of death, especially patients with high risk. Although this score can assist in the estimation of a prognosis in an individual patient after discharge as well as SE management, it still needs improvement and external validation.

## Supplementary information

**Additional file 1: **Causes of death and ICD-10 codes of predictors. **Figure S1.** Types of event. **Table S1.** Cause of death in each type of event. **Table S2.** ICD-10 codes of predictors

## Data Availability

The datasets used or analyzed during the current study are available from the corresponding author on reasonable request.

## Abbreviations

AUC: Area under the receiver operating characteristic curve
CI: Confidence interval
C-index: Concordance index
CNS: Central nervous system
EEG: Electroencephalogram
EMSE: Epidemiology-Based Mortality Score in Status Epilepticus
ICD-10: International Statistical Classification of Diseases and Related Health Problems 10th Revision
mRS: Modified Rankin Scale
mSTESS: Modified Status Epilepticus Severity Score
ROC: Receiver operating characteristic
SE: Status epilepticus
STESS: Status Epilepticus Severity Score
