# Supplementary material for: One- to 10-year Status Epilepticus Mortality (SEM) score after 30 days of hospital discharge: development and validation using competing risks analysis
Source: BMC Neurol. 2019 Dec 1;19:307. doi: 10.1186/s12883-019-1540-y (PMC6886213; doi:10.1186/s12883-019-1540-y)
Supplement: Supplementary file 1 — Additional file 1: Causes of death and ICD-10 codes of predictors. Figure S1. Types of event. Table S1. Cause of death in each type of event. Table S2. ICD-10 codes of predictors [file 12883_2019_1540_MOESM1_ESM.docx]

**Causes of death**

Direct causes from seizure, epilepsy, and status epilepticus

Accidents and suicides

Complications

of status epilepticus

Comorbidities

Other causes

Event of interest

Competing event

**Figure S1** Types of event

**Table S1** Cause of death in each type of event

| **Types of event** | **Cause of death** |
| --- | --- |
| **1. Event of interest** | |
| 1.1 Status epilepticus |  |
|  | Seizure |
|  | Status epilepticus |
| 1.2 Accidents and suicides |  |
|  | Aspiration |
|  | Burning |
|  | Drowning |
|  | Fall |
|  | Transport accident |
|  | Hanging |
|  | Self-poisoning |
| 1.3 SE complications |  |
|  | Acidosis |
|  | Acute renal failure |
|  | Airway obstruction |
|  | Anoxic brain damage |
|  | Brain death |
|  | Cardiac arrhythmias |
|  | Cardiorespiratory failure |
|  | Cerebral edema |
|  | Hyperglycemia |
|  | Hypoglycemia |
|  | Injuries of head |
|  | Pneumonia |
|  | Pulmonary edema |
|  | Respiratory failure |
|  | Status epilepticus complication (not identified) |
|  | Septicemia |
|  | Urinary tract infection |
| 1.4 Comorbidities |  |
|  | Alcohol abuse |
|  | Allergy |
|  | Anemia |
|  | Asthma |
|  | Brain tumor |
|  | Cancer |
|  | Cerebral palsy |
|  | Chronic kidney disease |
|  | Cirrhosis of liver |
|  | Comorbidities (not identified) |
|  | Deformations |
|  | Dementia |
|  | Diabetes |
|  | Encephalitis |
|  | Gout |
|  | Heart disease |
|  | Helminths of nervous system |
|  | Human immunodeficiency viruses disease |
|  | Hyperlipidemia |
|  | Hypertension |
|  | Hyperthyroidism |
|  | Intestinal obstruction |
|  | Muscle spasm |
|  | Parkinson's disease |
|  | Poliomyelitis |
|  | Pulmonary disease |
|  | Schizophrenia |
|  | Stroke |
|  | Systemic lupus erythematosus |
|  | Tuberculosis |
| **2. Competing events** | |
|  | Acute hepatic failure |
|  | Acute nephritic syndrome |
|  | Acute peritonitis |
|  | Assault |
|  | Cardiovascular collapse |
|  | Dengue fever |
|  | Disorders of mineral metabolism |
|  | Gastro-oesophageal laceration-haemorrhage |
|  | Hepatitis |
|  | Infectious causes |
|  | Malaria |
|  | Mosquito-borne viral encephalitis |
|  | Nutritional deficiencies |
|  | Senile |
|  | Shock |
|  | Sudden unexpected death syndrome |

**Table S2** ICD-10 codes of predictors

| **Predictors** | **ICD-10 code** |
| --- | --- |
| Brain tumor | C71, D33, D43 |
| Stroke | I60 to I69 |
| Epilepsy | G40 |
| Central nervous system infection | A06.6, A17, A39.0, A80 to A89, B00.3, B00.4, B01.0, B0.1.1, B02.0, B02.1, B05.0, B05.1, B06.0, B22.0, B26.1, B26.2, B37.5, B38.4, B43.1, B50.0, B58.2, B60.2, G00 to G09, R29.1 |
| Cancer  (not include brain tumor) | C00 to D48 (not include C71, D33, D43) |
| Diabetes | E10 to E14 |
| Hypertension | I10 to I15 |
| Chronic kidney disease | N18 |
| Heart disease | I05 to I09, I27, I30 to I52 |
| Ischemic heart disease | I20 to I25 |
| Shock | R57 |
| Septicemia | A40 to A41 |
| Hypoglycemia | E15, E16.0, E16.1, E16.2 |
| Pneumonia | J12 to J18 |
| Respiratory failure | J96 |
| Acute renal failure | N17 |
| Urinary tract infection | N39.0 |
